# Management of pregnancy at and beyond 41 completed weeks of gestation in low-risk women: a secondary analysis of two WHO multi-country surveys on maternal and newborn health

**DOI:** 10.1186/s12978-017-0394-2

**Published:** 2017-10-30

**Authors:** Kyaw Swa Mya, Malinee Laopaiboon, Joshua P. Vogel, Jose Guilherme Cecatti, João Paulo Souza, Ahmet Metin Gulmezoglu, Eduardo Ortiz-Panozo, Suneeta Mittal, Pisake Lumbiganon, on behalf of the WHO multi-country survey on maternal and newborn health research network

**Affiliations:** 1 Department of Epidemiology and Biostatistics, Faculty of Public Health, Khon Kaen University, Khon Kaen, 40002 Thailand; 2 Department of Biostatistics, University of Public Health, Yangon, Myanmar; 3 UNDP/UNFPA/UNICEF/WHO/World Bank Special Programme of Research, Development and Research Training in Human Reproduction (HRP), Geneva, Switzerland; 4 Department of Reproductive Health and Research World Health Organization, Geneva, Switzerland; 5 Department of Obstetrics and Gynecology, University of Campinas, Campinas, Brazil; 6 Department of Social Medicine, Ribeirão Preto Medical School, University of São Paulo, Ribeirão Preto, SP Brazil; 7 Center for Population Health Research, National Institute of Public Health, Cuernavaca, Mexico; 8 Department of Obstetrics & Gynecology, Fortis Memorial Research Institute, Gurgaon, India; 9 Department of Obstetrics and Gynaecology, Faculty of Medicine, Khon Kaen University, Khon Kaen, 40002 Thailand

**Keywords:** Prolonged pregnancy, Post-term pregnancy, Induction of labour, Elective caesarean section, Expectant management, Pregnancy outcomes

## Abstract

**Background:**

The World Health Organization (WHO) recommends induction of labour (IOL) for women who have reached 41 completed weeks of pregnancy without spontaneous onset of labour. Many women with prolonged pregnancy and/or their clinicians elect not to induce, and chose either elective caesarean section (ECS) or expectant management (EM). This study intended to assess pregnancy outcomes of IOL, ECS and EM at and beyond 41 completed weeks.

**Methods:**

This study is a secondary analysis of the WHO Global Survey (WHOGS) and the WHO Multi-country Survey (WHOMCS) conducted in Africa, Asia, Latin America and the Middle East. There were 33,003 women with low risk singleton pregnancies at ≥41 completed weeks from 292 facilities in 21 countries. Multilevel logistic regression model was used to assess associations of different management groups with each pregnancy outcome accounted for hierarchical survey design. The results were presented by adjusted odds ratios (aORs) with 95% confidence intervals (CIs) after adjusting for age, education, marital status, parity, previous caesarean section (CS), birth weight, and facility capacity index score.

**Results:**

The prevalence of prolonged pregnancy at facility setting in WHOGS, WHOMCS and combined databases were 7.9%, 7.5% and 7.7% respectively. Regarding to maternal adverse outcomes, EM was significantly associated with decreased risk of CS rate consistently in both databases i.e. (aOR0.76; 95% CI: 0.66–0.87) in WHOGS, (aOR0.67; 95% CI: 0.59–0.76) in WHOMCS and (aOR0.70; 95% CI: 0.64–0.77) in combined database, compared to IOL. Regarding the adverse perinatal outcomes, ECS was significantly associated with increased risks of neonatal intensive care unit admission (aOR1.76; 95% CI: 1.28–2.42) in WHOMCS and (aOR1.51; 95% CI: 1.19–1.92) in combined database compared to IOL but not significant in WHOGS database.

**Conclusions:**

Compared to IOL, ECS significantly increased risk of NICU admission while EM was significantly associated with decreased risk of CS. ECS should not be recommended for women at 41 completed weeks of pregnancy. However, the choice between IOL and EM should be cautiously considered since the available evidences are still quite limited.

## Plain English summary

Pregnancies beyond 41 completed weeks are associated with adverse outcomes. Hence, the World Health Organization recommends inducing labour for women who have reached 41 completed weeks of pregnancy without spontaneous labour pain. Many of such women and/or their clinicians do not want to induce, instead they prefer to deliver by caesarean section or expectant management (awaiting spontaneous onset of labour).

We compared pregnancy outcomes of women at 41 completed weeks of pregnancy by three different managements – induction of labour, elective caesarean section and expectant management using two large, WHO databases conducted in Africa, Asia, Latin America and the Middle East. We did not find any difference in adverse pregnancy outcomes between induction group and expectant group except higher caesarean section rate in induction of labour group.

We found that neonatal intensive care unit admission was higher in newborns delivered by elective caesarean section compared to that of induction of labour. Our findings showed that elective caesarean section had increased risk of adverse neonatal outcomes and should not be recommended. However, the choice between induction of labour and expectant management should be cautiously considered since the available evidences are still quite limited.

## Background

The World Health Organization (WHO) recommends induction of labour (IOL) for women who have reached 41 completed weeks of pregnancy without spontaneous onset of labour [1]. Rates of IOL vary across countries. IOL rates for high-income countries were 23.4% of deliveries in United States in 2010 [2], 22.1% of deliveries in England between 2011 and 2012 [3] and 25.4% of deliveries in Australia in 2010 [4]. The rates also vary for low and middle-income countries (LMIC). The WHO Global Survey on Maternal and Perinatal Health reported the prevalence of IOL in facility deliveries as 4.4% in seven African countries, 12.1% in nine Asian countries [5] and 11.4% in eight Latin American countries [6]. IOL is specifically recommended to prevent complications of prolonged pregnancy, such as increased perinatal mortality, stillbirth, fetal growth restriction, meconium aspiration syndrome and macrosomia [7–10]. However, IOL itself carries the risk of uterine hyperstimulation, increased instrumental delivery, uterine rupture, fetal distress and Caesarean section (CS) [1].

Many women with prolonged pregnancy (≥41 weeks) and/or their clinicians often elect not to induce, and chose either ECS or expectant management EM (awaiting spontaneous onset of labour). The reasons for choosing CS may be not only to manage the prolonged pregnancy, but also be the preferred mode of delivery for the women and/or the clinicians [11].

Systematic reviews of randomized controlled trials have compared the risks and benefits of IOL compared to EM at and beyond 41 weeks gestation [12–16]. All these systematic reviews assessed perinatal death and CS rate as primary outcomes, and other maternal and perinatal morbidities such as postpartum haemorrhage, ruptured uterus, meconium aspiration, Apgar score, NICU admission, stillbirth and early neonatal death as secondary outcomes. These reviews consistently reported that IOL at 41 completed weeks of gestation reduced the complications of postterm pregnancies compared to EM. However, the risks and benefits of IOL compared to ECS for women with prolonged pregnancy have not been as thoroughly explored. Furthermore, analysis of clinical data can provide insight into the effectiveness of interventions in “real life” settings. This analysis aimed to explore not only the risks and benefits of IOL but also that of ECS regarding to pregnancy outcomes among women with prolonged pregnancy in two large multi-country databases of facility deliveries in predominantly low- and middle-income countries.

## Methods

### Study design and setting

This study is a secondary analysis of two WHO databases: the WHO Global Survey (WHOGS) on Maternal and Perinatal Health [17] conducted in Africa, Asia and Latin America and the WHO Multi-country Survey (WHOMCS) on Maternal and Newborn Health conducted in Africa, Asia, Latin America and the Middle East [18]. WHOGS was conducted to explore the association between CS and maternal and perinatal outcomes in 286,565 women giving birth in373 facilities in 24 countries during 2004–2008. Building on the existing WHOGS network, the WHOMCS aimed to assess severe maternal and perinatal morbidity using the WHO maternal near-miss criteria in 314,623 women at 359 facilities in 29 countries during 2010–2011. All the participating countries except Japan and Qatar were in low and middle-income category (according to World Bank classifications) [19].

Details of the survey methods have been published elsewhere [17–20]. In brief, they were multi-centre, facility based, cross-sectional studies which used a stratified multistage cluster sampling method to select a sample of countries, provinces and health facilities. For the WHOGS, fourteen sub-regions from the six WHO regions were identified as the sampling frame. From each sub-region, four countries were randomly selected (probability proportional to population size). From each country, two provinces in addition to capital city were randomly selected. From each province, seven health facilities were randomly chosen from the health facilities with more than 1000 births per year and having ability to perform CS. In the WHOMCS, all WHOGS countries were invited to participate, however only 22 countries were able to participate. Two countries (Cuba and Algeria) were unable to participate. Within the remaining 22 countries, 32 facilities with very poor recruitment, data quality issues, or that were unable to participate were not included in the WHOMCS. Seven new countries (Afghanistan, Pakistan, Occupied Palestinian Territories, Mongolia, Jordan, Qatar and Lebanon) were added to improve global representation, bringing the total to 29 countries in WHOMCS.

Data in both surveys were collected using individual and institutional case record forms. Data were captured from the time the women first attended at facility for delivery until death, discharge or seventh postpartum day (whichever occurred first). Adverse pregnancy outcomes that occurred after discharge, during referral or after seventh postpartum day were not recorded. Data collection period was two months in health facilities with at least 6000 deliveries per year and three months in health facilities with less than 6000 deliveries per year. Socio-demographic characteristics, obstetric history, mode of delivery, labour characteristics and maternal and perinatal outcomes were collected for all women using pre-tested case record forms by trained data collectors. Health facility data concerning available obstetric and newborn services were also recorded in pre-tested institutional forms after consulting the head of department of obstetrics. Web-based data management systems were used for data entry of both databases. Internal consistency of data was randomly cross-checked comparing collected data and hospital records. The technical content of both protocols was reviewed by specialist panels at the UNDP/UNFPA/UNICEF/WHO/ World Bank Special Programme of Research, Development and Research Training in Human Reproduction. The Specialist Panel on Epidemiological Research reviewed and approved the WHOGS study protocol for technical content; the Research Project Review Panel (name of panel was changed in 2010) reviewed and approved the technical content of the WHOMCS.

**Fig. 1:**
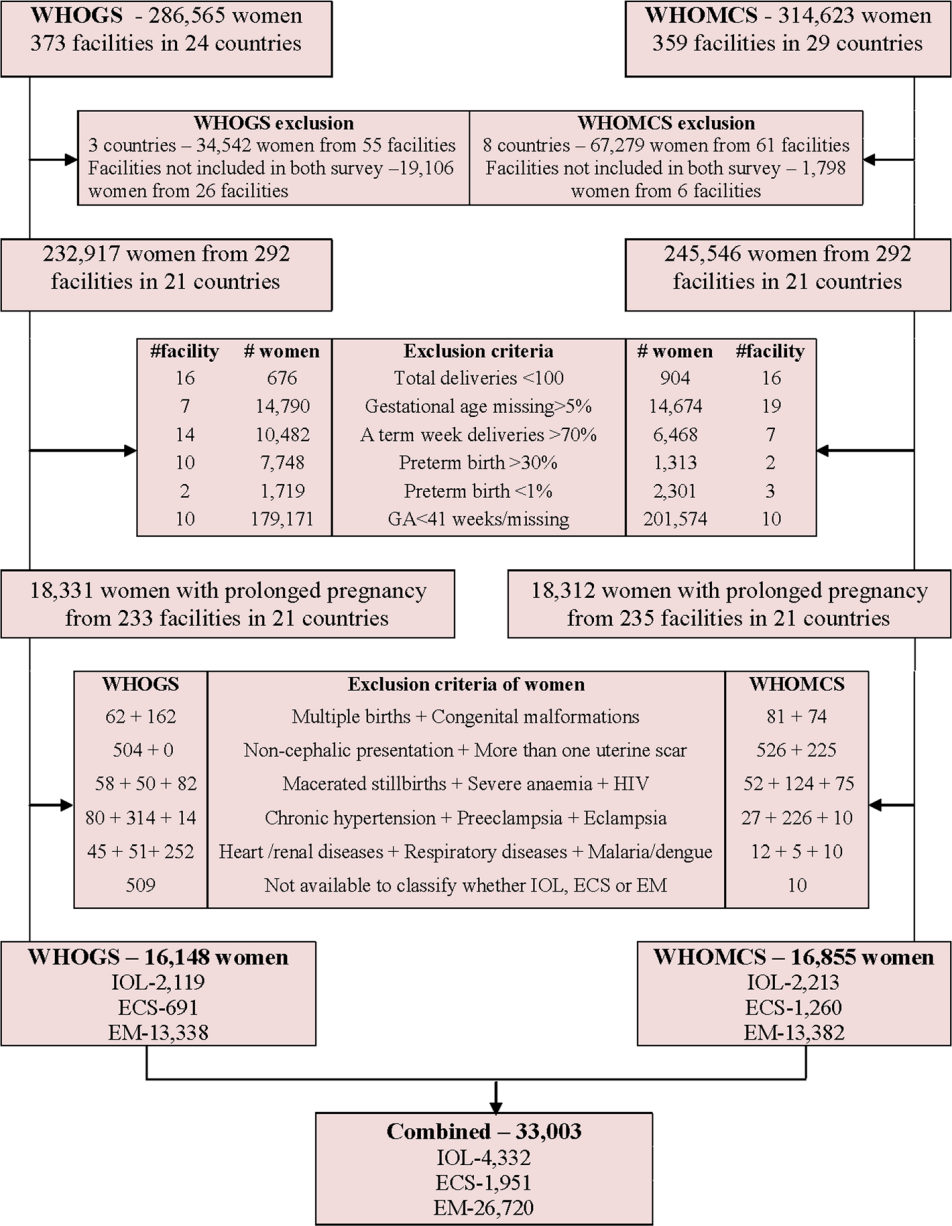
View of the scree parallel plot and scree simulation for determining the number of factors to retain

The WHOGS and the WHOMCS addressed different primary research questions, but both studies used a common list of core variables that enabled this secondary analysis. Some countries and facilities did not participate in both surveys; therefore, we restricted our analysis to the facilities and the countries that contributed data to both databases. Under this restriction, Cuba and Algeria from WHOGS and Afghanistan, Lebanon, Mongolia, Occupied Palestinian Territory, Pakistan, Qatar and Jordan from WHOMCS were excluded. Angola was excluded from this analysis due to concerns regarding poor data quality for gestational age (a critical variable for this analysis). A total of 21 countries participated in this analysis. Of these 21 countries 26 facilities from WHOGS and 6 facilities from WHOMCS were not included in both surveys, hence, excluded in this analysis. In addition, the facilities with unreliable information on gestational age distribution (i.e., facilities in which gestational age missing more than 5%, more than 70% of all deliveries occurred at a specific week, or where more than 30% or less than 1% of all deliveries were preterm) and with less than 100 total deliveries were excluded. Details on exclusion of women, facilities and countries are presented in Fig. 1. The current analysis has an analytical approach where three different procedures for managing low risk pregnancies at 41 completed weeks were evaluated in the same setting of 292 facilities in 21 countries.

### Study population

At the individual level, women with GA <41 weeks or missing data were excluded. A total of 18,331 women from 233 facilities of WHOGS and 18,312 women from 235 facilities of WHOMCS were identified as prolonged pregnancies (women with GA ≥ 41completed weeks of gestation). From this cohort of pregnant women, those with two or more previous CS, abnormal fetal presentation, pregnancy complications (preeclampsia, eclampsia), associated systemic diseases (heart disease, lung disease, renal disease, HIV/AIDS, malaria/dengue, severe anaemia (Hb < 7 g/L), and chronic hypertension) and women who delivered multiple births, babies with congenital malformation or macerated stillbirths were excluded. We excluded macerated stillbirths because they occurred before and were not consequences of IOL. Details are shown in Fig. 1.

In both surveys, we classified women using consistent definitions. Eligible women were classified into three groups based on the management at and beyond 41 completed weeks of gestation. Women who delivered their babies following IOL at 41 completed weeks were considered as the IOL group. Women of IOL group could deliver vaginally or by CS depending on whether the induction was successful or not. Women who delivered by ECS at 41 completed weeks in WHOGS and women delivered by prelabour CS at 41 completed weeks in WHOMCS were considered as ECS group. Women who delivered by spontaneous onset of labour at 41completed weeks and all women who delivered beyond 41 completed weeks (regardless of final mode of delivery) were considered as EM group. In this study, vaginal delivery included both normal and instrumental deliveries.

### Pregnancy outcomes and other variables

We classified pregnancy outcomes into maternal and perinatal outcomes. For maternal adverse outcomes we assessed postpartum haemorrhage (PPH), uterine rupture, admission to maternal ICU, maternal postpartum length of stay >7 days and severe maternal outcome. CS rate was also compared between IOL and EM groups. Postpartum haemorrhage, uterine rupture and severe maternal outcome were defined by different criteria for the two databases (as somewhat different variables were available on maternal outcomes). We presented these criteria for each database in Table 1.

**Table 1:** Criteria used to define maternal outcomes in WHOGS and WHOMCS databases

Maternal Outcomes	WHOGS Database	WHOMCS Database
Uterine rupture	Women who had laparotomy for uterine rupture or delivered by CS due to suspected/imminent uterine rupture	Women with complication of ruptured uterus
PPH	Women who had blood transfusion due to PPH or received uterotonics as a treatment of PPH	Women with complication of PPH
Severe maternal outcome	Women who had experienced in any of death or severe maternal morbidity – blood transfusion, hysterectomy, ICU admission and eclampsia	Women who had experienced maternal death or maternal near miss according to WHO criteria [20]

Perinatal adverse outcomes included stillbirth, early neonatal death, perinatal death, Apgar scores less than 7 at 5 min and admission to the neonatal intensive care unit (NICU).

Potential confounding factors were considered at both individual and facility levels. At the individual level, these included maternal age, education, marital status, parity, previous CS and birth weight. At the facility level, the availability of maternal healthcare services was classified into different levels, called facility capacity index (FCI) score. FCI score was calculated by using basic and essential services available at that facility and slightly different between WHOGS [21] and WHOMCS (due to differences in the institutional form used in the surveys) [22]. For consistency, we used FCI score calculated from WHOMCS in our analysis. It was ranged from 12 for the least resource service facility to 57 for the highest resource service facility. FCI score was used as a continuous variable and adjusted as a facility level covariate.

### Statistical analysis

We used frequency to describe maternal and neonatal characteristics of the three sub-populations for analysis in both surveys. Comparisons of maternal and neonatal characteristics among different management groups were done using chi-square test. Multilevel logistic regression model was used to assess associations of different management groups with each pregnancy outcome, adjusted for the potential confounding factors and hierarchical survey design, using two levels (individual and facility level) for separate analysis of WHOGS and WHOMCS. For the combined database, source of data (WHOGS and WHOMCS) was used as an additional level. Risks of maternal and neonatal adverse outcomes associated with each management group were assessed separately for WHOGS, WHOMCS and the combined database to identify the any consistency of associations. The results were presented by crude and adjusted odds ratios (crude ORs and AORs) with corresponding 95% confidence intervals (95% CIs). IOL group was used as a reference group because this is the procedure currently recommended by WHO [1]. Statistical analyses were done using lme4 package [23] in R software [24].

## Results

The prevalence of prolonged pregnancy at facility setting in WHOGS, WHOMCS and combined databases were 7.9% (18,331/232917), 7.5% (18,312/245546) and 7.7% (36,643/478463), respectively.

A total of 33,003 singleton pregnant women (16,148 women from WHOGS and 16,855 women from WHOMCS) with prolonged pregnancy (≥41 completed weeks) were included in this analysis. Median gestational age for this cohort was 41 and 95% CIs were 41–43 weeks. Amongst them, 13.1% (*n* = 4332) were delivered by IOL, 5.9% (*n* = 1951) were delivered by elective or prelabour CS and 80.9% (*n* = 26,720) were in EM group. Details are presented in Fig. 1.

The details of maternal and neonatal characteristics among the three different management groups for WHOGS and WHOMCS are presented in Table 2. We found that pregnant women were significantly different with respect to maternal age, education, parity, previous CS and newborns birth weight among the three different management groups in both WHOGS and WHOMCS but marital status was significantly different only in WHOGS.

**Table 2:** Maternal and neonatal characteristics of three different management groups for WHOGS and WHOMCS databases

Variables	WHOGS (*N* = 16,148) Different management groups			*P* value	WHOMCS (*N* = 16,855) Different management groups			*P* value
	IOL (*N* = 2119) *n* (%)	ECS (*N* = 691) *n* (%)	EM (N = 13,338) *n* (%)		IOL (*N* = 2213) *n* (%)	ECS (*N* = 1260) *n* (%)	EM (*N* = 13,382) *n* (%)	
Maternal characteristics								
Age (Years)								
≤ 19	168 (7.9)	53 (7.7)	1578 (11.8)	< 0.001	222 (10.0)	104 (8.3)	1532 (11.5)	0.003
20–34	1786 (84.3)	571 (82.6)	10,623 (79.7)		1801 (81.4)	1035 (82.1)	10,606 (79.3)	
≥ 35	165 (7.8)	67 (9.7)	1137 (8.5)		190 (8.6)	121 (9.6)	1244 (9.3)	
Education (School years)								
< 7	412 (20.0)	119 (17.3)	3218 (24.3)	< 0.001	290 (13.1)	195 (15.4)	2854 (21.3)	< 0.001
7–12	1173 (56.9)	409 (59.4)	7842 (59.3)		1151 (52.0)	728 (57.8)	7671 (57.3)	
> 12	476 (23.1)	161 (23.4)	2176 (16.4)		772 (34.9)	337 (26.8)	2857 (21.4)	
Marital								
With partner	1954 (92.3)	620 (90.0)	11,663 (87.5)	< 0.001	1944 (87.8)	1119 (88.8)	11,812 (88.3)	0.692
Without partner	164 (7.7)	69 (10.0)	1660 (12.5)		269 (12.2)	141 (11.2)	1570 (11.7)	
Parity								
Primiparous	1223 (57.7)	372 (53.8)	6353 (47.6)	< 0.001	1304 (59.0)	797 (63.3)	6496 (48.6)	< 0.001
Multiparous	896 (42.3)	319 (46.2)	6985 (52.4)		908 (41.0)	462 (36.7)	6879 (51.4)	
Previous CS								
Yes	43 (2.0)	171 (24.7)	731 (5.5)	< 0.001	75 (3.4)	259 (20.7)	879 (6.6)	< 0.001
No	2076 (98.0)	520 (75.3)	12,607 (94.5)		2135 (96.6)	993 (79.3)	12,480 (93.4)	
Neonatal characteristics								
Birth weight								
< 2500 g	57 (2.7)	9 (1.3)	359 (2.7)	< 0.001	71 (3.2)	33 (2.6)	448 (3.4)	< 0.001
2500–4000 g	1970 (93.0)	585 (84.7)	12,220 (91.6)		2017 (91.1)	1084 (86.0)	12,208 (91.2)	
> 4000 g	92 (4.3)	97 (14.0)	758 (5.7)		125 (5.7)	143(11.4)	726 (5.4)	

(*IOL* Induction of labour group, *ECS* Elective Caesarean section group, *EM* Expectant management group)

### Adverse maternal outcomes among different management groups

Table 3 showed comparison of adverse maternal outcomes among different management groups for WHOGS, WHOMCS and combined database, respectively. The association could not be assessed for ruptured uterus and ICU admission outcomes in WHOMCS as women with these outcomes were found only in EM group. In combined database, increased risk of ruptured uterus outcome was not statistically significant for EM compared to IOL (aOR 1.98; 95% CI: 0.51–7.65). EM was significantly associated with decreased risk of CS rate consistently in both databases i.e. (aOR0.76; 95% CI: 0.66–0.87) in WHOGS, (aOR0.67; 95% CI: 0.59–0.76) in WHOMCS and (aOR0.70; 95% CI: 0.64–0.77) in combined database, compared to IOL.

**Table 3:** Adverse maternal outcomes among different management groups in WHOGS, WHOMCS and Combined databases

Adverse maternal outcomes	WHOGS			WHOMCS			Combined		
	n/N (%)	Crude OR^a^ (95% CI)	AOR^b^ (95% CI)	n/N (%)	Crude OR^a^ (95% CI)	AOR* (95% CI)	n/N (%)	Crude OR^a^ (95% CI)	AOR^b^ (95% CI)
PPH									
IOL	88/2119 (4.2)	1	1	38/2213 (1.7)	1	1	126/4332 (2.9)	1	1
ECS	11/691 (1.6)	0.78 (0.39, 1.56)	0.66 (0.30, 1.44)	19 /1260 (1.5)	1.01 (0.54, 1.87)	0.99 (0.50, 1.98)	30 /1951 (1.5)	1.01 (0.66, 1.53)	0.89 (0.56, 1.43)
EM	542/13338 (4.1)	1.12 (0.84, 1.51)	1.21 (0.86, 1.70)	173/13382 (1.3)	0.88 (0.58, 1.33)	0.91 (0.57, 1.45)	715/26720 (2.7)	1.03 (0.82, 1.29)	1.03 (0.80, 1.33)
Ruptured uterus									
IOL	3/2119 (0.5)	1	1	0/2213 (0.0)	–	–	3/4332 (0.1)	1	1
ECS	2/691 (0.3)	0.65 (0.10, 4.04)	0.40 (0.05, 3.22)	0 /1260 (0.0)	–	–	2 /1951 (0.1)	0.74 (0.15, 3.60)	0.48 (0.07, 3.05)
EM	40/13338 (1.2)	2.70 (0.79, 9.28)	1.52 (0.32, 7.16)	10/13382 (0.1)	–	–	50/26720 (0.3)	**3.29 (1.16, 9.33)**	1.98 (0.51, 7.65)
Admission to ICU									
IOL	24/2119 (1.1)	1	1	0/2213 (0.0)	–	–	24/4332 (0.6)	1	1
ECS	5/691 (0.7)	1.85 (0.58, 5.88)	1.17 (0.35, 4.03)	0 /1260 (0.0)	–	–	5 /1951 (0.3)	1.21 (0.49, 2.96)	1.09 (0.36, 3.31)
EM	258/13338 (1.9)	0.62 (0.37, 1.03)	0.60 (0.34, 1.03)	8/13382 (0.1)	–	–	266/26720 (1.0)	0.69 (0.44, 1.07)	0.66 (0.39, 1.13)
Postpartum length of stay >7 days									
IOL	76/2119 (3.6)	1	1	44/2213 (2.0)	1	1	120/4332 (2.8)	1	1
ECS	21/691 (3.0)	**2.18 (1.29, 3.68)**	1.22 (0.68, 2.19)	49 /1260 (3.9)	**2.33 (1.51, 3.60)**	1.54 (0.96, 2.49)	70 /1951 (3.6)	**2.16 (1.57, 2.99)**	1.33 (0.93, 1.90)
EM	373/13338 (2.8)	1.08 (0.80, 1.45)	0.98 (0.71, 1.35)	424/13382 (3.2)	0.81 (0.57, 1.15)	0.83 (0.57, 1.21)	797/26720 (3.0)	0.91 (0.73, 1.13)	0.87 (0.69, 1.11)
Severe maternal outcome^c^									
IOL	52/2119 (2.5)	1	1	4/2213 (0.2)	1	1	56/4332 (1.3)	1	1
ECS	14/691 (2.0)	1.41 (0.73, 2.70)	1.15 (0.57, 2.34)	3 /1260 (0.2)	1.41 (0.30, 6.63)	0.64 (0.10, 3.97)	17 /1951 (0.9)	1.42 (0.83, 2.46)	1.08 (0.59, 1.96)
EM	382/13338 (2.9)	0.80 (0.56, 1.14)	0.86 (0.59, 1.27)	18/13382 (0.1)	0.67 (0.21, 2.13)	0.55(0.16, 1.79)	400/26720 (1.5)	0.77 (0.56, 1.04)	0.80 (0.57, 1.13)
CS rate									
IOL	590/2119 (27.8)	1	1	690/2213 (31.2)	1	1	1280/4332 (29.6)	1	1
EM	3416/13338 (25.6)	**0.82 (0.73, 0.92)**	**0.76 (0.66,0.87)**	3463/13382 (25.9)	**0.69 (0.61, 0.77)**	**0.67 (0.59,0.76)**	6879/26720 (25.7)	**0.74 (0.68, 0.80)**	**0.70 (0.64,0.77)**

(*IOL* Induction of labour group, *ECS* Elective Caesarean section group, *EM* Expectant management group) ^a^Crude ORs were calculated by multilevel logistic regression model after accounted for cluster effect (facility level and country level) ^b^Adjusted – for maternal characteristics (age, marital status, education, parity, previous CS); neonatal characteristics (birth weight); facility capacity index score and cluster effect (health facility level, country level in WHOGS and WHOMCS; one more level (data source – WHOGS & WHOMCS) in combined database ^c^Severe maternal outcome – presence of at least one of maternal death, blood transfusion, hysterectomy, eclampsia and admission to ICU in WHOGS; presence of maternal death or maternal near miss in WHOMCS (Maternal near miss is defined as WHO criteria)Values in bold mean they are statistically significant (*p* < 0.05)

### Adverse perinatal outcomes among different management groups

Use of ECS was significantly associated with increased risks of NICU admission, aOR 1.76; 95%CI: 1.28–2.42 in WHOMCS and aOR 1.51; 95%CI: 1.19–1.92 in combined database, compared to IOL. The association was not statistically significant in WHOGS database. Apart from NICU outcome, the rest of perinatal outcomes were not significantly associated with different managements groups. Details are presented in Table 4.

**Table 4:** Adverse perinatal outcomes among different management groups in WHOGS, WHOMCS and Combined databases

Adverse perinatal outcomes	WHOGS			WHOMCS			Combined		
	n/N (%)	Crude OR^a^ (95% CI)	AOR^b^ (95% CI)	n/N (%)	Crude OR^a^ (95% CI)	AOR^b^ (95% CI)	n/N (%)	Crude OR^a^ (95% CI)	AOR^b^ (95% CI)
APGAR <7 at 5 min									
IOL	40/2119 (1.9)	1	1	23/2213 (1.1)	1	1	63/4332 (1.5)	1	1
ECS	3/691 (0.4)	0.39 (0.12, 1.22)	0.31 (0.08, 1.23)	13/1260 (1.0)	1.20(0.61, 2.36)	0.92 (0.45, 1.90)	16/1951 (0.8)	0.81 (0.47, 1.40)	0.67 (0.36, 1.23)
EM	309/13338 (2.3)	1.10 (0.77, 1.57)	1.21 (0.82, 1.81)	232/13382 (1.8)	1.41 (0.91, 2.20)	1.39 (0.88, 2.19)	541/26720 (2.0)	1.19 (0.90, 1.57)	1.27 (0.95, 1.72)
Admission to NICU									
IOL	146/2119 (6.9)	1	1	101/2213 (4.6)	1	1	247/4332 (5.7)	1	1
ECS	67/691 (9.7)	**1.61 (1.15, 2.26)**	1.13 (0.75, 1.72)	117 /1260 (9.3)	**1.99 (1.47, 2.71)**	**1.76 (1.28, 2.42**)	184/1951 (9.4)	**1.85 (1.49, 2.31)**	**1.51 (1.19, 1.92)**
EM	1035/13338 (7.8)	0.94 (0.76, 1.15)	0.99 (0.79, 1.26)	587/13382 (4.4)	0.90 (0.71, 1.15)	0.92 (0.72, 1.18)	1622/26720 (6.1)	0.93 (0.80, 1.08)	0.95 (0.81, 1.12)
Stillbirth									
IOL	10/2119 (0.5)	1	1	8/2213 (0.4)	1	1	18/4332 (0.4)	1	1
ECS	0/691 (0.0)	–	–	4 /1260 (0.3)	1.06 (0.31, 3.62)	0.99 (0.27, 3.57)	4/1951 (0.2)	0.70 (0.24, 1.99)	0.61 (0.20, 1.83)
EM	47/13338 (0.4)	0.69 (0.33, 1.46)	0.63 (0.29, 1.38)	73/13382 (0.6)	1.29 (0.60, 2.78)	1.29 (0.56, 2.98)	120/26720 (0.5)	0.97 (0.58, 1.60)	0.92 (0.54, 1.58)
Early neonatal death									
IOL	2/2119 (0.1)	1	1	7/2213 (0.3)	1	1	9/4332 (0.2)	1	1
ECS	1/691 (0.1)	2.25 (0.20, 25.5)	2.10 (0.18, 24.7)	4 /1260 (0.3)	1.23 (0.35, 4.35)	0.95 (0.26, 3.45)	5/1951 (0.2)	1.69 (0.59, 4.85)	1.35 (0.46, 4.00)
EM	50/13338 (0.4)	4.17 (0.99, 17.5)	3.82 (0.89, 16.2)	67/13382 (0.5)	1.28 (0.57, 2.91)	1.11 (0.48, 2.56)	117/26720 (0.4)	1.94 (0.99, 3.80)	1.74 (0.88, 3.45)
Perinatal death									
IOL	12/2119 (0.6)	1	1	15/2213 (0.7)	1	1	27/4332 (0.6)	1	1
ECS	1/691 (0.1)	0.42 (0.06, 3.02)	0.41 (0.05, 3.10)	8 /1260 (0.6)	1.15 (0.50, 2.66)	0.96 (0.40, 2.30)	9/1951 (0.5)	1.06 (0.50, 2.22)	0.90 (0.42, 1.94)
EM	97/13338 (0.7)	1.27 (0.68, 2.36)	1.18 (0.62, 2.22)	139/13382 (1.0)	1.20 (0.70, 2.05)	1.14 (0.65, 2.02)	236/26720 (0.9)	1.26 (0.83, 1.89)	1.19 (0.77, 1.82)

(*IOL* Induction of labour group, *ECS* Elective Caesarean section group, *EM* Expectant management group) ^a^Crude ORs were calculated by multilevel logistic regression model after accounted for cluster effect(facility level and country level) ^b^Adjusted – for maternal characteristics (age, marital status, education, parity, previous CS); neonatal characteristics (birth weight); facility capacity index score and cluster effect (health facility level, country level in WHOGS and WHOMCS; one more level (data source – WHOGS & WHOMCS) in combined database Values in bold mean they are statistically significant (*p* < 0.05)

One consideration in this analysis is that some women in the EM group may experience induction and/or CS at a later gestational age. To account for this, we re-classified those women in the EM group (at 41 completed weeks) who experienced induction or ECS later in pregnancy (i.e. beyond 41 completed weeks). There were 1759 pregnant women in the EM group with deliveries at beyond 41 weeks of gestation. They could be classified to IOL group for 1217 women and ECS group for 542 women. We did sensitivity analyses of the associations of the new classification of management groups with each pregnancy outcome in the combined database. The results showed consistent findings with our main analysis. In addition the sensitivity analyzed results provided stronger associated with increased risk of postpartum length of stay >7 days outcome (aOR1.59; 95% CI: 1.17–2.18) and admission to NICU outcome (aOR1.54; 95% CI: 1.25–1.90) for ECS compared to IOL. The stronger associated with decreased risk of severe maternal outcome (aOR0.73; 95% CI: 0.55–0.99), rate of CS (aOR0.65; 95% CI: 0.59–0.70) and admission to NICU outcome (aOR0.84; 95% CI: 0.72–0.97) were also seen for EM compared to IOL. Details are presented in Table 5. We also assessed the risk of stillbirths by week delivered using 41 weeks as a reference group. The risk was not significantly different at 42 weeks (aOR1.47; 95% CI: 0.82–2.62). However, as gestational age reached 43, 44 and 45 weeks, the risk of stillbirth were significantly increased, aOR3.45; 95% CI: 1.32–9.03 for 43 weeks, aOR 6.15; 95% CI: 1.38–27.48 for 44 weeks and aOR18.04; 95% CI: 1.96–166.11 for 45 weeks. Details are presented in Table 6.

**Table 5:** Adverse maternal and perinatal outcomes among different management groups in combined databases (Sensitivity analysis)

	Combined		
	n/N (%)	Crude OR^a^ (95% CI)	AOR^b^ (95% CI)
Adverse maternal outcomes			
PPH			
IOL	168/5549 (3.03)	1.0	1.0
ECS	45/2493 (1.81)	0.98 (0.69, 1.40)	0.90 (0.61, 1.33)
EM	658/24,961 (2.64)	0.87 (0.71, 1.06)	0.88 (0.70, 1.11)
Ruptured uterus			
IOL	5/5549 (0.14)	1.0	1.0
ECS	2/2493 (0.08)	0.42 (0.09, 1.79)	0.21 (0.04, 1.06)
EM	48/24,961 (0.31)	2.72 (1.18, 6.25)	1.25 (0.45, 3.45)
Admission to ICU			
IOL	32/5549 (0.58)	1.0	1.0
ECS	9/2493 (0.36)	2.06 (1.01, 4.21)	1.56 (0.65, 3.76)
EM	254/24,961 (1.02)	0.78 (0.52, 1.15)	0.72 (0.45, 1.14)
Postpartum stay > 7			
IOL	154/5549 (2.78)	1.0	1.0
ECS	101/2493 (4.05)	2.48 (1.87, 3.29)	1.59 (1.17, 2.18)
EM	732/24,961 (2.93)	0.97 (0.79, 1.18)	0.92 (0.74, 1.15)
Severe maternal outcome			
IOL	79/5549 (1.42)	1.0	1.0
ECS	24/2493 (0.96)	1.50 (0.94, 2.40)	1.18 (0.71, 1.96)
EM	370/24,961 (1.48)	0.72 (0.54, 0.94)	0.73 (0.55, 0.99)
CS rate			
IOL	1591/5549 (28.67)	1.0	1.0
EM	6026/24,961 (24.14)	0.70 (0.65, 0.75)	0.65 (0.59, 0.70)
Adverse perinatal outcomes			
APGAR <7 at 5 min			
IOL	109/5549 (1.97)	1.0	1.0
ECS	29/2493 (1.17)	0.86 (0.56, 1.30)	0.67 (0.41, 1.08)
EM	482/24,961 (1.94)	0.88 (0.70, 1.10)	0.97 (0.76, 1.24)
Admission to NICU			
IOL	344/5549 (6.22)	1.0	1.0
ECS	257/2493 (10.31)	1.89 (1.57, 2.29)	1.54 (1.25, 1.90)
EM	1452/24,961 (5.83)	0.82 (0.72, 0.94)	0.84 (0.72, 0.97)
Stillbirth			
IOL	25/5549 (0.45)	1.0	1.0
ECS	7/2493 (0.28)	0.91 (0.40, 2.08)	0.76 (0.30, 1.91)
EM	110/24,961 (0.44)	0.94 (0.60, 1.48)	1.01 (0.62, 1.65)
Early neonatal death			
IOL	23/5549 (0.42)	1.0	1.0
ECS	7/2493 (0.28)	0.91 (0.39, 2.08)	0.71 (0.30, 1.68)
EM	101/24,961 (0.41)	0.93 (0.59, 1.48)	0.82 (0.51, 1.32)
Perinatal death			
IOL	48/5549 (0.87)	1.0	1.0
ECS	14/2493 (0.56)	0.93 (0.51, 1.69)	0.75 (0.39, 1.43)
EM	210/24,961 (0.84)	0.92 (0.66, 1.27)	0.90 (0.63, 1.27)

*IOL* Induction of labour group, *ECS* Elective Caesarean section group, *EM* Expectant management group

^a^Crude ORs were calculated by multilevel logistic regression model after accounted for cluster effect(facility level, country level, data source -WHOGS & WHOMCS)

^b^Adjusted for maternal characteristics (age, marital status, education, parity, previous CS); neonatal characteristics (birth weight); facility capacity index score and cluster effect (facility level, country level, data source – WHOGS & WHOMCS)

**Table 6:** Risk of stillbirth by week delivered

Stillbirth	Combined		
	n/N (%)	Crude OR^a^ (95% CI)	AOR^b^ (95% CI)
41 completed week	95/25,631 (0.37)	1.0	1.0
42 completed week	32/6335 (0.51)	1.15 (0.76, 1.71)	1.47 (0.82, 2.62)
43 completed week	10/829 (1.21)	2.91 (1.52, 5.58)	3.45 (1.32, 9.03)
44 completed week	3/157 (1.91)	4.36 (1.39, 13.64)	6.15 (1.38, 27.48)
45 completed week	2/51 (3.92)	11.12 (2.72, 45.41)	18.04 (1.96, 166.11)

^a^Crude ORs were calculated by multilevel logistic regression model after accounted for cluster effect (facility level, country level, data source -WHOGS & WHOMCS)

^b^Adjusted for maternal characteristics (age, marital status, education, parity, previous CS); neonatal characteristics (birth weight); facility capacity index score and cluster effect (facility level, country level, data source – WHOGS & WHOMCS)

## Discussion

The prevalence of prolonged pregnancy was about 8% of total deliveries. The prevalence of prolonged pregnancy varies across the world from 3 to 12% [25] and our analysis supports those findings. The prevalence is affected by accuracy of gestational age estimation and adoption of elective induction of labour before 41 weeks of gestation [8]. The risk of stillbirth increased significantly when gestational age was 43 weeks or over compared to 41 weeks.

ECS was significantly associated with increased risk of adverse perinatal outcomes compared to IOL for NICU admission in WHOMCS and combined databases. Compared to IOL, EM was significantly associated with decreased risk of CS consistently in all three databases.

Four previous systematic reviews [12, 14–16] reported the effect of IOL compared to EM among prolonged/post-term pregnancies. Systematic reviews of Wennerholm et al., 2009 [15] and Sanchez-Ramos et al., 2003 [16] reported no significant difference in perinatal deaths while those of Gülmezoglu et al., 2012 (Cochrane review) [12] and Hussain et al., 2011 [14] reported significantly lower perinatal death in IOL group. Three systematic reviews i.e. Sanchez-Ramos et al., Wennerholm et al. and Gülmezoglu et al. reported significant lower CS delivery rate in IOL compared to EM. These reviews support the WHO recommendation of routine use of IOL for pregnancies at 41 completed weeks. Our analysis of routinely collected data showed no significant difference in perinatal deaths, however there was a significant lower CS rate in EM compared to IOL in all databases. Possible explanations include: (1) in the Cochrane review, number of perinatal deaths were very small (only 10 deaths) but perinatal death rate of EM was 8 times more than IOL (0.32% vs. 0.04%). In our analysis, perinatal death was more common (272 deaths) but perinatal death rate of EM was only 1.4 times more than IOL (0.88% vs. 0.62%), (2) our results were from hierarchical databases and we adjusted for multiple potential confounders and clustering effects of facility and country levels in the analysis, this could lead to more conservative confidence interval and (3) the number of participants in the Cochrane review were quite similar across comparison groups but that of our study groups were quite different in number. One more explanation for the difference between our findings and the systematic reviews may relate to difference in context. Trials are generally conducted in highly controlled, often ideal settings (and often in high-resource settings), which may favour efficacy. Our data relates more to real-life practices (i.e. effectiveness) in resource-limited settings, where the intervention may not be as beneficial as trials might suggest. A recently published before and after study comparing a policy induction of labour at 41 weeks versus at 42 weeks showed a significantly lower CS rate, 15% versus 19.4% (*p* = 0.0135)in a 41-week policy. There were no significant differences in maternal or neonatal outcomes [26].

In spite of our extensive literature search, we did not identify any studies comparing IOL and ECS for women at or beyond 41 completed weeks of gestation. Similar with our results, previous study conducted among women at all gestational ages also showed that CS increased the risk of maternal and neonatal adverse outcomes [17].

To our knowledge, this analysis was the first report of the adverse pregnancy outcomes of ECS among women with prolonged pregnancy. This study was based on two large WHO databases conducted in Africa, Asia, Latin America and the Middle East regions. Data were systematically collected by trained personnel. Thus the results of this study reflect the actual practices in participating facilities and their pregnancy outcomes in these facilities.

However, the WHO databases were collected for other specific objectives rather than to explore our study question. Moreover, the two surveys used slightly different case record forms to collect individual and institutional characteristics. Consequently, a few adverse outcomes (postpartum haemorrhage, uterine rupture and severe maternal outcome) had slightly different definitions. Despite this, we elected to combine the databases and analyzed these outcomes collectively as we regarded the outcomes as sufficiently similar.

Both databases were facility based, conducted mainly in larger, secondary and tertiary facilities where CS was available – this might lead to over-representation of adverse outcomes among women and their newborns. Thus, this study results might not be representative in smaller or different facilities. The primary data source was routine hospital records; these may not be ideal in many facility settings. Some facilities encountered suboptimal record collection such as lack of documented diagnosis. It may be due to inability to diagnose condition, failure to recognize condition or failure to document diagnosis, rather than absence of condition. To address this, several facilities adopted the study data collection form as a platform for their medical records. In the WHOMCS, in cases of unclear or missing information, medical staffs were asked to clarify information in the medical record.

The gestational age used was the best obstetric estimate based on local practices. The method of GA assessment was unknown; but usually included the calculation from last menstrual period and/or ultra-sonographic examination. Inaccurate estimate of gestational age can lead to over-estimate prolonged pregnancy [27] and thus may have affected the results of this analysis. However, this misclassification would bias the risk assessment toward unity. The risk that we have estimated should be more conservative. We also tried to minimize the effect of gestational age inaccuracy on this analysis by excluding facilities with GA missing >5% and with unreliable information on gestational age distribution such as more than 70% of all deliveries occurred at a specific week, or where more than 30% or less than 1% of all deliveries were preterm (Fig. 1). Furthermore, data concerning with methods, types of drugs and routes of administration for IOL cervical ripening methods, oxytocin augmentation protocols, use of partogram, use of electronic fetal monitoring and epidural analgesia rates were not available in databases, so, we could not adjust for these variations to evaluate the outcomes of IOL group.

The differences in institutional intrapartum practices might influence the pregnancy outcomes. We used multilevel logistic regression model to account this effect and hierarchical survey design, using two levels (individual and facility level) for separate analysis of WHOGS and WHOMCS. Moreover, two WHO surveys conducted at different time period and the intrapartum practices of the same facilities might change in two surveys. We accounted for this effect, source of data (WHOGS and WHOMCS) as an additional level for the analysis in combined database.

In addition we did the sensitivity analyses and it showed similar associations among the three management groups when some women were reclassified based on their management beyond 41 weeks of gestation. The other limitation of this study is that it was hospital based and didn’t have information on those women who didn’t come to the hospital for delivery.

## Conclusions

Compared to IOL, ECS significantly increased risk of NICU admission while EM was significantly associated with decreased risk of CS. ECS should not be recommended for women at 41 completed weeks of pregnancy without spontaneous pain. However, the choice between IOL and EM should be cautiously considered since the available evidences are still quite limited.

## Abbreviations

AIDS: Acquired Immune Deficiency Syndrome
CS: Caesarean Section
ECS: Elective Caesarean Section
EM: Expected Management
GA: Gestational age
HIV: Human Immunodeficiency Virus
IOL: Induction of Labour
UNDP: United Nations Development Programme
UNFPA: United Nations Population Fund
UNICEF: United Nations International Children’s Emergency Fund
WHO: World Health Organization
WHOGS: World Health Organization Global Survey
WHOMCS: World Health Organization Multicountry Survey
